# Utilizing Latent Dirichlet Allocation and Differential Abundance to Identify Microbial Communities in both the Oral and Fecal Microbiome Associated with Alzheimer’s Disease

**DOI:** 10.1002/alz.089296

**Published:** 2025-01-03

**Authors:** Ziyuan Huang, Abigail L Zeamer, Doyle Ward, Cynthia Jo, Vanni Bucci, John P Haran

**Affiliations:** ^1^ UMass Chan Medical School, Worcester, MA USA; ^2^ University of Massachusetts Chan Medical School, Worcester, MA USA; ^3^ University of Massachusetts Chan Medical School Chan Medical School, Worcester, MA USA

## Abstract

**Background:**

Several studies have found that oral and gut microbiome and their byproducts can impact Alzheimer’s Disease (AD). The objective of our study is to analyze metagenomic sequencing data from paired oral and fecal microbiomes, along with clinical variables, to identify communities of bacteria associated with AD. This research aims to improve our understanding of the microbiome community matrix, and how these communities interact and correlate with AD status compared to healthy controls (HC) through an oral‐gut microbial axis.

**Method:**

The study includes 223 HC and 43 individuals with AD. During each visit, paired oral and fecal samples were collected, along with clinical variables. Metagenomic profiling was done on all samples. Latent Dirichlet Allocation (LDA) was applied to identify differences in microbial species groups between these two body sites in realtion to AD status. LDA is used as a topic modeling method to uncover the complex structure and function of microbial communities. Subsequently, differential abundance (DA) analysis was performed to identify species with differential abundance at each body site.

**Result:**

We identified microbiotal communities sharing similar characteristics and pinpointed representative bacteria within these communities that are highly relevant to AD. Within the oral microbiome, we have identified 27 topics, including several bacteria that are highly relevant to AD. These included *Alistipes* (beta = 3.919232e‐01), *Paraprevotella xylaniphila* (beta = 1.227791e‐01), *Desulfovibrio* (beta = 6.013213e‐02), and *Lachnospiraceae* (beta = 2.304369e‐02). In the gut, we have identified 50 topics, reflecting the gut is more complex the oral microbiome. Notable bacteria in the gut microbiome include *Actinomyces oricola* (beta = 6.959554e‐01), *Roseburia* (beta = 8.861444e‐02), *Bacteroidetes* (beta = 5.010610e‐01), and *Actinomyces gerencseriae* (beta = 3.048668e‐02).

**Conclusion:**

Our study has identified a variety of bacteria that exhibit novel community patterns that associate with AD. In the gut, *A. gerencseriae* and other oral microbiomes were observed in AD patients. Also, the microbial communities differ between AD and HC. Thereforth, we conclude that translocation of oral and gut microbiota may contribute to AD through an oral‐gut‐microbiome axis.

